# Professionals' and Intercultural Mediators' Perspectives on Communication With Ukrainian Refugees in the Czech Healthcare System

**DOI:** 10.1111/hex.14171

**Published:** 2024-08-16

**Authors:** Jolana Kopsa Těšinová, Karolína Dobiášová, Marie Jelínková, Elena Tulupova, Michal Koščík

**Affiliations:** ^1^ Institute of Public Health and Medical Law, First Faculty of Medicine Charles University Prague Czech Republic; ^2^ Institute of Social Sciences, Faculty of Social Sciences Charles University Prague Czech Republic; ^3^ Department of Public Health, Faculty of Medicine Masaryk University Brno Czech Republic

**Keywords:** communication barriers, forced migration, health care, intercultural mediators, Ukrainians

## Abstract

**Introduction:**

A growing body of research is examining how healthcare systems are responding to the increasing numbers of migrants and the resulting superdiversity of patients. The aim of this article is to identify and explain communication barriers in the provision of healthcare to Ukrainian war refugees in the Czech Republic from the perspectives of healthcare professionals and intercultural mediators.

**Methods:**

The exploratory case study is based on a qualitative analysis of semi‐structured interviews with frontline health professionals: 20 with doctors and 10 with nurses. The second source of data is two focus groups aimed at capturing communication problems from the perspective of intercultural mediators who accompany refugees to health facilities. The interview transcripts and FGs were analysed using six‐stage thematic coding.

**Results:**

The survey identified five main themes related to barriers to communication: (1) language barriers and interpreting, (2) cultural barriers, (3) differing expectations of health and the healthcare systems in the Czech Republic and Ukraine, (4) prejudices and negative attitudes and unethical behaviour towards refugees and migrants and (5) lack of awareness of patient rights.

**Conclusions:**

The arrival of large numbers of migrants has highlighted deficiencies in the system that may affect other vulnerable groups and the general population. These include the lack of general communication skills and legal awareness among many health professionals, which are barriers to the development of patient‐centred care. The involvement of intercultural mediators fundamentally improves communication between health professionals and (not only) migrant patients. Nevertheless, it is necessary to legally anchor and define the position of intercultural mediators within the healthcare system.

**Patient or Public Contribution:**

Collaboration with intercultural mediators who interpreted the extensive experiences of Ukrainian refugee patients and also have personal experience as migrant or migrant‐origin patients contributed to shaping research questions, facilitating study participation and enriching evidence interpretation. Researchers with multicultural backgrounds and experience with working with people from refugee backgrounds were involved in the study design and analysis.

## 1 Introduction

The Russian military invasion of Ukraine on 24 February 2022 triggered the largest refugee crisis in Europe since the Second World War [1]. The Czech Republic took in more than 550,000 Ukrainian war refugees in a short period of time, with approximately 390,000 currently residing in the Czech Republic [2]. By law, all of these refugees have access to healthcare under the same conditions as Czech citizens [3], with most healthcare expenses covered by compulsory public health insurance and low patient financial co‐payments (11.5% of all expenses) [4]. This has significantly increased the number of patients from different cultural backgrounds and with limited knowledge of the Czech language, which has placed increased demands on Czech doctors and nurses, especially in the area of communication.

Communication in healthcare provision not only affects patient satisfaction and adherence but also healthcare outcomes [5]. In this context, a major challenge for the Czech healthcare system is intercultural communication between health professionals and patients with a migrant background, where different perspectives, values and beliefs about illness and healthcare can lead to misunderstandings and difficulties in providing healthcare, which can subsequently affect the quality of the care [5, 6, 7]. Therefore, the World Health Organization emphasizes the need to identify communication barriers in the provision of healthcare to migrants—including refugees—so that healthcare systems can respond to them with appropriate interventions [8, 9]. Communication barriers and their solutions in the provision of healthcare to refugees have received considerable attention in the professional literature [6, 10]. A number of studies from different countries show that communication barriers can be a cause of health inequalities [7].

The aim of this article is to identify and explain communication barriers in the provision of healthcare to Ukrainian war refugees in the Czech Republic from the perspective of healthcare professionals and intercultural mediators.

## 2 Materials and Methods

### 2.1 Study Design

This study is part of a broader research project focused on the availability of healthcare for Ukrainian war refugees in the Czech Republic. Considering this previously unexplored topic, a qualitative methodological approach in the form of an exploratory case study was chosen [11]. In the first phase of the research, we conducted semi‐structured interviews with healthcare professionals who are so‐called frontline workers. These were doctors and nurses from among (a) general practitioners for adults at outpatient clinics (hereafter GP), (b) general practitioners for children and adolescents at outpatient clinics (hereafter GPCA), (c) hospital emergency departments and (d) low‐threshold outpatient clinics for Ukrainian refugees (UA POINT) who had the experience of providing healthcare services to Ukrainian refugees. UA POINTS were established in all teaching hospitals by decision of the Minister of Health after the outbreak of the war in Ukraine and the arrival of the first wave of Ukrainian refugees [12]. In the second phase of the research, we focused on the perspective of intercultural mediators who had the experience of accompanying Ukrainian refugee patients within the Czech healthcare system, with whom two focus groups (FGs) were conducted. The ‘intercultural mediator’ (also named intercultural worker, intercultural assistant, community interpreter) is a bilingual community worker, usually with a migrant background, working mostly for nongovernmental organizations and a municipal government [13]. An intercultural mediator provides individual support to migrants and refugees in dealing with public institutions, including interpreting, mediation and conflict prevention, and assists organizations in providing culturally sensitive care [14].

### 2.2 Data Collection and Research Sample

The first source of data is 20 interviews with doctors and 10 interviews with nurses, ranging in duration from 30 to 120 min. Most of the interviews were conducted in person, with a few conducted online via the Zoom platform. The interviews took place between September 2022 and June 2023. Interviews were conducted by team members based on a pre‐prepared interview script. The interview script was structured into several thematic blocks: introduction of the respondent, experiences of healthcare professionals with Ukrainian refugees and other migrants, accessibility of healthcare for Ukrainian refugees, communication, healthcare professional–patient relationships with a migrant background and intercultural education of doctors and nurses. Informants were selected using a purposive sampling method [15] so that the sample included a variety of respondents in terms of the type of care provided, region, gender and length of experience of doctors and nurses. The resulting sample of informants is shown in Tables 1 and 2.

**Table 1:** Characteristics of medical informants—doctors.

Informant identification	Gender	Length of work experience (years)	Healthcare facility
Doctor 1	Female	12	GP
Doctor 2	Female	15	GP
Doctor 3	Male	10	GP
Doctor 4	Male	37	GP
Doctor 5	Male	23	GP
Doctor 6	Male	2	Hospital, UA POINT, hospital emergency
Doctor 7	Male	28	GP
Doctor 8	Male	40	GPCA
Doctor 9	Female	20	GPCA
Doctor 10	female	4.5	Hospital emergency
Doctor 11	Male	3	Hospital emergency
Doctor 12	Male	2	GP
Doctor 13	Male	32	Hospital emergency for children
Doctor 14	Female	24	GPCA
Doctor 15	Female	28	GPCA and UA POINT
Doctor 16	Male	4	Hospital emergency
Doctor 17	Male	2	Hospital emergency
Doctor 18	Male	2	Hospital emergency
Doctor 19	Male	6	Hospital emergency for children
Doctor 20	Male	26	Hospital emergency

**Table 2:** Characteristics of medical informants—nurses.

Informant identification	Gender	Length of work experience (years)	Healthcare facility
Nurse 1	Female	30	UA POINT
Nurse 2	Female	12	GP office
Nurse 3	Female	32	Hospital emergency room
Nurse 4	female	7	Hospital emergency room
Nurse 5	Female	4	Hospital emergency room
Nurse 6	Female	20	Hospital emergency room for children
Nurse 7	Female	28	UA POINT for children
Nurse 8	Female	32	Hospital emergency room for children
Nurse 9	Female	39	GP office
Nurse 10	Female	25	GP office

The second source of data are FGs with intercultural mediators. All mediators were Ukrainian/Russian speakers, as languages understandable for majority of Ukrainian refugees include Ukrainian and/or Russian. The FG method allowed us to make use of spontaneously occurring group interactions during discussions [16, 17]. The topics for discussion were prepared in advance by the research team. FG1 was conducted online so that intercultural mediators from regions of the country other than the capital city of Prague could participate. FG2 was conducted in person, and involved intercultural mediators from Prague (FG2); both FGs took place in June 2023. In each FG, one member of the research team with migration experience moderated the group discussion (E.T.), whereas the other (K.D.) participated in the role of ‘silent observer’, that is, sporadically entering the discussion and noting the group dynamics, the general atmosphere of the discussion and other characteristics of the participants [11]. The characteristics of the FG participants are presented in Table 3.

**Table 3:** FG participant characteristics.

FG participant identification	Gender	Languages used in which intercultural work was provided
Focus Group 1		
IKM1‐1	Female	Russian
IKM1‐2	Female	Ukrainian
IKM1‐3	Female	Ukrainian, Russian
IKM1‐4	Female	Russian
IKM1‐5	Female	Ukrainian, Russian
IKM1‐6	Male	Russian
IKM1‐7	Female	Russian
Focus Group 2		
IKM2‐1	Female	Ukrainian, Russian
IKM2‐2	Female	Russian
IKM2‐3	Female	Ukrainian
IKM2‐4	Female	Russian
IKM2‐5	Female	Russian

### 2.3 Ethical Considerations

The research was approved by the Ethics Committee of the General University Hospital in Prague, No. 205/22 S‐IV. All informants were informed about the purpose and meaning of the research and the voluntary nature of participation in the research, and signed an informed consent form. The interviews and FGs were recorded, transcribed verbatim and anonymized.

### 2.4 Data Analysis

The interview transcripts and FGs were analysed using six‐stage thematic coding [18]. In the initial stage of analysis, the researchers (J.K.T. and K.D.) re‐read all interview and FG transcripts and extracted sections of text relating to communication between healthcare professionals and refugee patients. Subsequently, all team members thoroughly familiarized themselves with the data. During the familiarization process, the FG field notes were also studied. In the second phase, the data were independently coded by three of the authors (J.K.T., K.D. and M.J.) so that each occurrence had its own separate code. Subsequently, the codes were compared and refined after repeated discussions amongst themselves (J.K.T., K.D. and M.J.) to organize the data and categorize the emerging underlying themes. Once the core themes were identified, further discussions and revisions of themes took place among the researchers within the entire multidisciplinary team until all authors agreed that the final revised themes were representative of the data presented in the coded data. In the final step, six major themes were identified and named, which are presented in the following section of this paper. Manual coding was used for analysis. The data were managed in Word and Excel.

## 3 Results

The survey identified five main themes related to barriers to communication: (1) language barriers and interpreting, (2) cultural barriers, (3) differing expectations of health and the healthcare systems in the Czech Republic and Ukraine, (4) prejudices and negative attitudes and unethical behaviour towards refugees and migrants and (5) lack of awareness of patient rights.

### 3.1 Language Barrier and Interpretation

Most Czech healthcare professionals perceived the lack of knowledge of the Czech language among Ukrainian refugee patients as a major problem in communication. Exceptionally, some of the interviewed doctors (not nurses) communicated with Ukrainian refugees partly in Russian or English, but the use of various translation software (most often Google Translate) was a more common practice for communication. The use of informal interpreters—Ukrainian employees of healthcare institutions—was also a frequent solution, especially in hospitals. Before the outbreak of the war in Ukraine, as of 31 December 2021, 196,000 Ukrainians were registered in the Czech Republic, more than 80% of whom were working [19]. The employment of Ukrainian migrants in the healthcare sector in less skilled jobs was frequent [20]. Doctors and nurses reflected that this solution was not the most appropriate.

Sometimes, the problem with communication is that Ukrainian patients have an advantage over other foreign patients in that a large part of the lower staff in hospitals […] is also of Ukrainian nationality, so there is almost always someone on the ward who can interpret. But it is not always advisable to have a cleaning lady interpreting …

(D17)

Interpreting by family members of Ukrainian patients (including children) and acquaintances was also frequent. Interpreting by a formal interpreter or intercultural mediator was sporadically used. Exceptionally, an interpreter on the phone was used.

We don't have an official interpreter; we mainly use Google Translate.

(D16)

Some health professionals mentioned cases where patients arrived without an interpreter, and the lack of (or no) Czech language skills on the part of refugees, along with the lack of Ukrainian or Russian language skills on the part of health professionals, led to difficulties in communication and limited information provided by Ukrainian patients. This lack of communication made it difficult to make a diagnosis and prescribe appropriate treatment.

They can't manage to converse with the doctor on the same level as a Czech person, who can ask more questions about things that interest and concern them. A refugee from Ukraine prefers to say ‘yes’ or ‘no’, and nod to anything, just to avoid communicating.

(D19)

Reflection on the difficulty of linguistic understanding varied substantially between doctors and nurses. Those who had more extensive experience with foreigners already had established ways of communicating. For health professionals who had only sporadically treated foreigners before the arrival of Ukrainian refugees, language communication was a significant complication in the provision of care.

With Ukrainians who do not have interpreters, and where we do not understand each other, it is difficult.

(D9)

Doctors appreciated the various medical history questionnaires in Russian and Ukrainian, along with translations and a list of common medications in Ukraine provided by the Ministry of Health, medical societies and NGOs, as a great aid to communication.

We have a Facebook group of GPs where we share different experiences. So, for example, we shared some documents or anamnesis questionnaires in Ukrainian, or we wrote about the vaccination situation in Ukraine, etc.

(D2)

Another barrier often mentioned by health professionals in the communication with refugees was the greater time required for treatment.

It's very time‐consuming. I usually take ten minutes to examine a patient if they come in for a check‐up. But then one foreigner comes in who doesn't understand, and that takes half an hour, even if they come with a problem that I would normally resolve in five minutes.

(D2)

From the point of view of both health professionals and intercultural mediators, communication by telephone or email, as well as making appointments through systems that are only available in Czech, emerged as a highly problematic area for refugees within the health system.

The telephone communication is very poor. The language barrier is much worse there. They think the only way they can get to us is by coming straight to us, which, of course, is a complication, because nowadays we use a 100% ordering system.

(D4)

Intercultural mediators noted that doctors and nurses do not have experience in using professional interpreters and intercultural mediators and often do not know what their areas of competence are. Similarly, intercultural mediators described situations when doctors approach them as if they were healthcare workers.

Doctors are already used to this, and I can see that they appreciate when an intercultural mediator accompanies a patient. Sometimes, however, when it comes to professional vocabulary, doctors think that the intercultural mediator is a medical expert …

(IKM1‐5)

Intercultural mediators described encountering misunderstandings of their roles by healthcare professionals, which leads to doctors not respecting the mediator's right not to be present during a medical procedure while providing interpretation.

I explain to them that I'm not a doctor, that I can interpret, but I don't need to witness the procedure. That I'll stay behind that tarp or turn my back. And he (the doctor): ‘No, no, turn around and look at this.’ They must also respect the intercultural worker, that we are not doctors and we do not need to witness the procedure like they do.

(IKM2‐5)

Intercultural mediators also cited situations where the failure to provide an interpreter by Ukrainian refugees was a reason for refusing to provide healthcare.

When a refugee comes in alone, they are usually refused: ‘Come back with an interpreter.’

(IKM2‐3)

### 3.2 Cultural Barriers

The Czech Republic and Ukraine are both Slavic countries, and their culture and languages are partly similar. Moreover, before the outbreak of the war, Ukraine was the main source country for economic migrants to the Czech Republic, and so a significant number of healthcare professionals had had experience with Ukrainian economic migrants.

Nevertheless, a significant proportion of healthcare professionals perceived certain cultural differences in communicating with refugees, compared with Czech patients, and this was often a source of discomfort.

… the patient simply comes in thinking they have to fight to get care … and they think they need to yell and scream.

(D1)

They want everything it and now. And then they escalate the aggression and one has to stand there and explain.

(N10)

In particular, nurses who receive patients for care reported that this aggressive behaviour is often a source of conflict.

Many times they are arrogant, that they want to go straight to the doctor and I am in the way.

(N10)

In this context, uncertainty was expressed by both doctors and nurses about how to act in such situations.

I don't know what to say, how to react, how to handle them, what to use so that I'm not the one who gets triggered, but to respond in a way that they accept it and realize that I'm not attacking them, and we're not going to attack each other.

(N3)

Most of the interviewed healthcare professionals mentioned that they had received little or no training in intercultural education.

This information (intercultural education) was completely lacking in pre‐ and postgraduate education. They are not included in any curriculum of pre‐ or postgraduate education.

(D11)

### 3.3 Different Expectations in Health and Healthcare Systems in the Czech Republic and Ukraine

Most of the doctors, nurses and intercultural mediators shared experiences where many of the problems in communication between healthcare professionals and refugee patients were caused by the manifest differences in the healthcare systems of the Czech Republic and Ukraine.

Healthcare professionals here in the Czech Republic do not understand the healthcare system that exists in Ukraine … They do not understand the reason why, for example, Ukrainian refugees call an ambulance. In Ukraine, they use ambulances like an intermediate level of GP.

(IKM2‐5)

Both healthcare professionals and intercultural mediators agreed that, in this context, it would be useful to explain the functioning of the Czech healthcare system to Ukrainian refugees.

There is a real need for someone to explain the Czech medical system to them. They are used to completely different conditions.

(D15)

Interestingly, in connection with the differences in the Ukrainian healthcare system, most of the doctors interviewed would appreciate if they could also be trained in how the healthcare system works there.

As to the different functioning of the healthcare systems in the two countries, doctors in particular often pointed out the dissimilar expectations in the relationship between the doctor and a Ukrainian refugee patient. Doctors repeatedly reported experiences where Ukrainian refugees came in with their own ideas about diagnosis and treatment, and they expected the doctor to treat them according to their instructions. The attitude of Ukrainian patients reflects the fact that, due to the limited functioning of the Ukrainian healthcare system, patients there are more used to self‐managing their treatment [21, 22]. In Ukraine, the existence of direct (often informal) payments for the healthcare provision, medical supplies and medicines also fundamentally affects the doctor–patient relationship [22]. This attitude of Ukrainian patients was often assessed by doctors as unpleasant, and perceived as a lack of respect for their expertise.

They come in and they're already dictating to me what to do, which annoys us as doctors.

(D4)

I had a terribly hard time explaining to them that they just can't have the colonoscopy immediately on demand, even if they want to pay the 5,000 CZK, they just have to queue up like any other patient.

(D15)

Another problem mentioned was the different approach of the Czech and Ukrainian healthcare systems to the prescription of medicines and medical devices. In Ukraine, prescriptions are not regulated to the same extent as in the Czech Republic, and private pharmacies are known to be very liberal in dispensing medicines and medical devices there [21, 22]. The different expectations of Ukrainian patients regarding the availability of medicines and medical devices led to a number of misunderstandings in practice.

They want to get an injection in the first place, receive antibiotics. They are not used to leaving the doctor's office without an injection or antibiotics.

(N8)

### 3.4 Negative Attitudes and Unethical Behaviour

The majority of healthcare professionals reported that the arrival of Ukrainian refugees brought an increased workload to their practices, caused not only by the higher number of patients but also by their ‘otherness’, for which they were not prepared. This has led to negative attitudes towards refugees among some healthcare professionals regarding in particular possible abuses of care.

Refugees are different because at least 75% of them come purely deliberately. […]But it's just calculated; they can't afford it at home because over there they have to pay for healthcare, and here they know they don't have to, so they take advantage of it. […]It seems to me that they want to milk the system to death.

(N1)

The same experience was noted by the intercultural workers.

(Some doctors) say that they (Ukrainian patients) will come here and start using our medicines. They are annoyed that they're taking advantage of health insurance that is basically free for them. I've heard that really a lot of times.

(IKM2‐3)

Intercultural workers also described the relatively common experience of inappropriate communication among nurses about Ukrainian patients, even in their presence or in the presence of intercultural mediators. The intercultural workers perceived this as very unethical behaviour.

But the nurses, they dare to talk among themselves about these people, and these people clearly know that they are talking about them, even though they may not understand them. And that's just not ethical and it's not normal behavior.

(IKM2‐2)

### 3.5 Lack of Awareness of Patients' Rights

In the Czech Republic, the provision of health care is based on the principle of the patient's freedom and autonomy of will, and medical intervention for a person who is capable of deciding their own fate can essentially be rendered only with their informed consent. This is in contrast to a paternalistic approach, where decisions that concern a patient's health are made by others, even if for the right motives. Patient education requires not only the fulfilment of legal obligations in terms of its scope and form but also a certain degree of empathy (soft skills).

In contrast, intercultural workers noted that not enough attention is paid by health professionals to explaining the follow‐up care needed, which does not reflect the position of war refugees and their complex orientation in the Czech health system. The lack of confirmation of understanding on the part of Ukrainian patients is also a problem.

… some doctors do not fulfill their information obligation towards the refugee patient, refuse to explain the specifics of treatment, do not confirm that the patient understands, upon leaving, what to do next … We encounter ignorance and misunderstanding that Ukrainians are not informed about the system.

(IKM1‐5)

Intercultural workers also pointed out that in the case of extensive translation about a procedure associated with potentially serious risks to the patient's life or health, the doctors did not provide enough time not only for translation but also for the understanding of the patient.

I had to translate some complications before a surgical procedure, and that was terribly stressful. If something happened, it would be on my account for not translating something. And there were four pages of those complications. I read and translate the complications quickly, and there has to be time for that.

(IKM2‐5)

## 4 Discussion

Communication is essential for building trust between healthcare professionals and patients from culturally and linguistically diverse backgrounds [23]. Conversely, language and communication barriers, lack of trust and perceived discrimination may increase the risk of unmet needs and poorer quality of care among refugees [24, 25]. The results of our research showed, similar to other studies [26, 27], that the language barrier is the biggest source of problems in the daily practice of doctors and nurses in providing care to Ukrainian war refugees. That is the cause of poor communication and lack of information sharing. Similarly, in a survey by the European Union Agency for Fundamental Rights (FRA) [28], war refugees from Ukraine most frequently cited the language barrier as a barrier to accessing healthcare in the Czech Republic (49%). In this context, a number of research studies [26] have shown that language is a key factor affecting refugees' overall experience of healthcare. If this experience is negative, it can reinforce a pre‐existing sense of alienation from the host society and increase refugees' levels of social exclusion, negatively impacting on how they use health services [25, 29].

Informants among healthcare professionals agreed that care for refugees is significantly more time‐consuming than for Czech patients due to the language barrier. This may in practice be a source of inadequate provision of care [25]. Another problem area related to the language barrier, according to both health professionals and intercultural mediators, is communication using information and communication technologies, including making appointments with doctors using appointment systems that are mostly available only in Czech [30, 31]. Furthermore, our research has shown that the language barrier between Czech health professionals and Ukrainian refugees is often overcome in practice by ad hoc solutions such as informal translators and interpreters from family and Ukrainian‐ or Russian‐speaking staff in health facilities. Health professionals themselves reflected on the risks of using these approaches, which is consistent with findings that informal interpreting can be a source of miscommunication or breach of confidentiality [32, 33]. Although guidelines and toolkits exist in different countries for deciding what type of interpreter health professionals should use in a particular case [34], Czech doctors and nurses do not have such methodologies available, and their choice of communication strategies is influenced by practical and financial circumstances [32].

In contrast to healthcare professionals, intercultural mediators have repeatedly observed situations where Ukrainian refugee patients were denied care in cases where they did not have an interpreter or intercultural mediator with them, despite the fact that under Czech legislation, it is the duty of the health provider to ensure that the patient is informed about his/her health condition in an understandable way, even if he/she is a foreigner [35]. Our findings are in line with the results of the FRA [28] survey, where 10% of Ukrainian refugees surveyed had experienced denial of healthcare in the Czech Republic.

Healthcare professionals pointed out that communication with Ukrainian patients is complicated by many factors other than language, such as lack of trust or sociocultural differences between refugees and the majority. Other research has documented similar experiences [36]. The doctors and nurses had encountered behaviour with Ukrainian refugees that they were not used to. A frequently mentioned problem from the side of health professionals was aggression from refugees and demanding immediate care. Similar experiences of healthcare staff in providing care to refugees have been reported in other European studies [37, 38], where healthcare professionals gave examples of conflict situations in which refugee patients behaved aggressively.

On the other hand, intercultural mediators noted instances where Czech health professionals showed negative attitudes and unethical behaviour towards refugees, associated with a certain stereotyping of Ukrainian refugees as someone who abuses the Czech healthcare system. The experience of refugees facing prejudice and sometimes discriminatory behaviour from health professionals is also reported in other studies [26, 39]. Negative attitudes towards refugees are linked to the inability of health professionals to manage the ‘otherness’ [39].

Moreover, the intercultural mediators pointed to situations where Czech healthcare professionals did not demonstrate basic communication skills when communicating with refugees, did not know the rights of patients and practiced a paternalistic approach, despite the fact that the Czech Republic defines the rights of patients to participate in treatment in accordance with the Convention on Human Rights and Biomedicine. The development of communication skills is not a mandatory part of the education of health professionals in the Czech Republic. The lack of general basic communication skills is a major limitation to any intercultural communication in the case of a vulnerable refugee group [5].

A number of misunderstandings in communication in our research were related to the different functioning of the Czech and Ukrainian healthcare systems. Ukrainian refugees came with the experience of patients as being much more involved in their treatment, both in terms of care management and payment [21]. Different expectations about prescribing medication were also a frequently mentioned cause of misunderstandings. Similar experiences have been reported in research by Haj‐Younes et al. [25], where refugees, for example, had higher expectations of antibiotic prescription based on their country‐of‐origin experiences. According to Bardby et al. [40], health workers should be able to explain the healthcare system in the host country while having a basic understanding of the specific situation in the migrant's country of origin. They should be able to recognize and manage misunderstandings caused by cultural differences and different patient expectations of the healthcare system [5].

In order for the complex needs of refugees to be met by the healthcare system, it is essential that health workers have adequate training in intercultural competence [41]. For the successful development of intercultural competence in healthcare workers, not only knowledge and skills in relation to refugees but also changing their attitudes towards refugees are crucial [42]. Thus, it is also important to develop intercultural sensitivity in education [43], which has a positive impact on changing health workers' attitudes towards refugees [44]. A number of expert studies [29, 45, 46, 47] point out that the promotion and development of cultural competence and training in intercultural communication skills should be an integral part of the training curriculum for all categories of healthcare providers [32].

However, most of the informants testified that they were not adequately prepared during their undergraduate or postgraduate training to provide care to refugees and immigrants, including practicing conflict resolution. Despite the long‐term increase in the number of foreigners in the Czech Republic, intercultural education of healthcare professionals is not part of the standard curriculum in the Czech Republic [48].

In addition to intercultural education, the involvement of intercultural mediators is an important element of responsive healthcare and contributes to reducing conflicts [49] and advocates for individual patients or patient groups, particularly their rights and interests when discriminatory practices limit access to—or quality of—care [50]. With the arrival of Ukrainian refugees, the work of intercultural mediators, who accompany refugee patients to healthcare facilities, became more prevalent in the country. However, the intercultural mediators pointed out that health professionals often do not know their competences and rights and, last but not least, confuse them with professional interpreters. The reason for this is that intercultural mediators are not a permanent/standard part of the Czech healthcare system; their role is not defined in the healthcare legislation. Their services are, as in other countries, often offered within various short‐term and non‐systematic projects and programmes [32, 33]. In the Czech Republic, like in some other countries receiving Ukrainian war refugees [51], there are not enough intercultural mediators.

## 5 Conclusion

The results of our research have shown that the arrival of a huge number of war refugees from Ukraine in a relatively short period of time caught the Czech healthcare system unprepared, especially for the linguistic and cultural differences of refugee patients. In view of the increasing diversity of patients, it will be necessary to build a system of intercultural education for all healthcare professionals as an integral part of the standard curriculum. However, the arrival of a large number of patients who are different from the general population and show a greater degree of vulnerability has also highlighted deficits in the system that may affect other vulnerable groups and the general population. These include the lack of acquisition and application of general communication skills and legal awareness among many health professionals, which are barriers to the development of patient‐centred care. To improve communication between health professionals and patients (not only) with a migration background, it will also be necessary to legally anchor and define the position of intercultural mediators within the health system. Interventions aimed at increasing the awareness of Ukrainian refugees about the functioning of the Czech healthcare system and the involvement of trusted representatives of this community in these interventions will also be necessary.

In terms of our research methodology, the involvement of intercultural mediators proved beneficial. Thanks to the experience of intercultural mediators accompanying Ukrainian refugees and their experience with the functioning of the Czech healthcare system, we were able to capture a number of communication problems that could not be detected by the ‘mere’ perspective of healthcare professionals or refugees themselves. Nevertheless, recently arrived Ukrainians were not directly involved in this research; their involvement in future research is desirable.

The arrival of Ukrainian refugees can be seen as an opportunity for reform steps to address deficits in the training of health professionals and the functioning of the healthcare system in order to improve care not only for vulnerable populations.

## Author Contributions

**Jolana Kopsa Těšinová:** data curation, conceptualization, methodology, validation, visualization, writing–review and editing, project administration, formal analysis, software, supervision, funding acquisition. **Karolína Dobiášová:** conceptualization, investigation, writing–original draft, methodology, validation, writing–review and editing, formal analysis, software, supervision, data curation. **Marie Jelínková:** writing–original draft, methodology, validation, formal analysis, resources, investigation, conceptualization. **Elena Tulupova:** validation, writing–review and editing, resources. **Michal Koščík:** validation, data curation, project administration.

## Ethics Statement

The research was conducted in accordance with the ethical principles of sociological research and was approved by the Ethics Committee of the General University Hospital in Prague (No.: 205/22 S‐IV).

## Conflicts of Interest

The authors declare no conflicts of interest.

## Data Availability

Data from the study are available in full upon reasonable request in accordance with local ethics board guidelines. The anonymized transcripts of all interviews and focus groups were conducted in the Czech language.
